# Long-term changes in habitat and trophic level of Southern Ocean squid in relation to environmental conditions

**DOI:** 10.1038/s41598-020-72103-6

**Published:** 2020-09-16

**Authors:** José Abreu, Richard A. Phillips, Filipe R. Ceia, Louise Ireland, Vítor H. Paiva, José C. Xavier

**Affiliations:** 1 Department of Life Sciences, University of Coimbra, Mare—Marine and Environmental Sciences Centre, 3000-456 Coimbra, Portugal; 2 British Antarctic Survey, Natural Environment Research Council, High Cross, Madingley Road, Cambridge, CB3 0ET UK

**Keywords:** Climate-change ecology, Stable isotope analysis, Ecology, Ecology

## Abstract

Long-term studies of pelagic nekton in the Southern Ocean and their responses to ongoing environmental change are rare. Using stable isotope ratios measured in squid beaks recovered from diet samples of wandering albatrosses *Diomedea exulans*, we assessed decadal variation (from 1976 to 2016) in the habitat (*δ*^13^C) and trophic level (*δ*^15^N) of five important Southern Ocean squid species in relation to indices of environmental conditions—Southern Oscillation Index (SOI) and Southern Annular Mode (SAM). Based on *δ*^13^C values, corrected for the Suess effect, habitat had changed over the last 50 years for *Taonius* sp. B (Voss), *Gonatus antarcticus*, *Galiteuthis glacialis* and *Histioteuthis atlantica* but not *Moroteuthopsis longimana*. By comparison, mean *δ*^15^N values were similar across decades for all five species, suggesting minimal changes in trophic levels. Both SAM and SOI have increased in strength and frequency over the study period but, of the five species, only in *Taonius* sp. B (Voss) did these indices correlate with, *δ*^13^C and *δ*^15^N values, indicating direct relationships between environmental conditions, habitat and trophic level. The five cephalopod species therefore changed their habitats with changing environmental conditions over the last 50 years but maintained similar trophic levels. Hence, cephalopods are likely to remain important prey for top predators in Southern Ocean food webs, despite ongoing climate change.

## Introduction

Environmental conditions have a major influence on the structure and function of ecological systems^1–3^. The Southern Ocean has shown extensive environmental changes in recent decades and will continue to do so^3–5^. The two leading modes of environmental variability in the region—the Southern Oscillation Index (SOI) and the Southern Annular Mode (SAM)—have grown in strength and frequency, associated with more and stronger El Niño events and increasing intensity of westerly winds^6–8^. Both SOI and SAM have tangible climatic influences on biological processes^2,9^. El Niño events are characterized by warmer waters in the south Pacific, which generate anomalies in sea surface temperature in the Southern Ocean^10,11^. These changes mainly affect primary production, and are associated with reduced abundance and recruitment of Antarctic krill *Euphasia superba* (hereafter krill) in the Scotia Sea, including around South Georgia^12–14^. Additionally, different phases of the SAM are associated with changes in the direction and intensity of the westerly wind system^7^, which drives water circulation in the Southern Ocean, influencing chlorophyll concentrations and upwelling intensities^15,16^.

The ability of organisms to adapt to this periodic environmental perturbation has repercussions for their performance and survival, and hence has a major influence on population dynamics^17–21^. Depending on different factors, changing conditions may favour particular individuals, populations or species. Many studies have examined responses to changing oceanographic conditions of organisms at different trophic levels, or sought to predict the impacts on distributions^22,23^. In the Southern Ocean, documented effects of climate variability on ecological processes, include changes in diet, foraging areas, breeding success or abundance, particularly at higher trophic levels, such as in penguins and albatrosses^24–27^. The only such studies on key pelagic zooplankton or nekton are those on krill^28,29^. However, nekton such as squid are also major components in the ecosystem^30–33^. These taxa may be well adapted to environmental perturbation, and hence their importance could increase relative to others in the food web^23,34,35^.

Squid have a high turn-over rate because of their short life-spans (1–2 years because of extensive post-spawning mortality)^36^. This gives them the capacity to respond faster than other species to climate change or an increase in fishing pressure^35^. They are mostly pelagic, feeding on a variety of fish, other cephalopods and crustaceans, shifting from crustaceans to fish, and hence in trophic level, during their life^37,38^. Squid occur across different water masses in the Southern Ocean and are key prey of a wide range of predators including whales, seals, seabirds and fishes, with some 34 million tonnes consumed annually^30^. The predator that probably takes by far the greatest diversity (~ 50 species) of squid is the wandering albatross *Diomedea exulans*, which is therefore an effective biological sampler of squid in the Southern Ocean^39,40^. Prior to fledging, wandering albatross chicks regurgitate a bolus (pellet) of indigestible material, including squid beaks, which has accumulated over the previous 8–9 months since hatching^41^. Stable isotope ratios of carbon (^13^C/^12^C; δ^13^C) and nitrogen (^15^N/^14^N; δ^15^N) in these beaks can provide invaluable information on, respectively, the carbon source (habitat) and trophic position^37,42,43^.

Understanding changes in habitat and trophic levels of squid provides unique and valuable insights both into the state of the pelagic system in the Southern Ocean in past decades, and its likely future response to scenarios of predicted environmental change^3,44,45^. Within this context, the objectives of this study were to: (1) use changes in stable isotope ratios in squid beaks to assess variation over the last five decades (from the mid-1970s to 2010s) in the habitat and trophic level of key squid species in the Southern Ocean, (2) determine the influence of oceanographic conditions on this variation, and (3) discuss the implications for how squid may cope with future environmental change in the region.

## Results

### Decadal variation in stable isotope ratios

**Figure 1:**
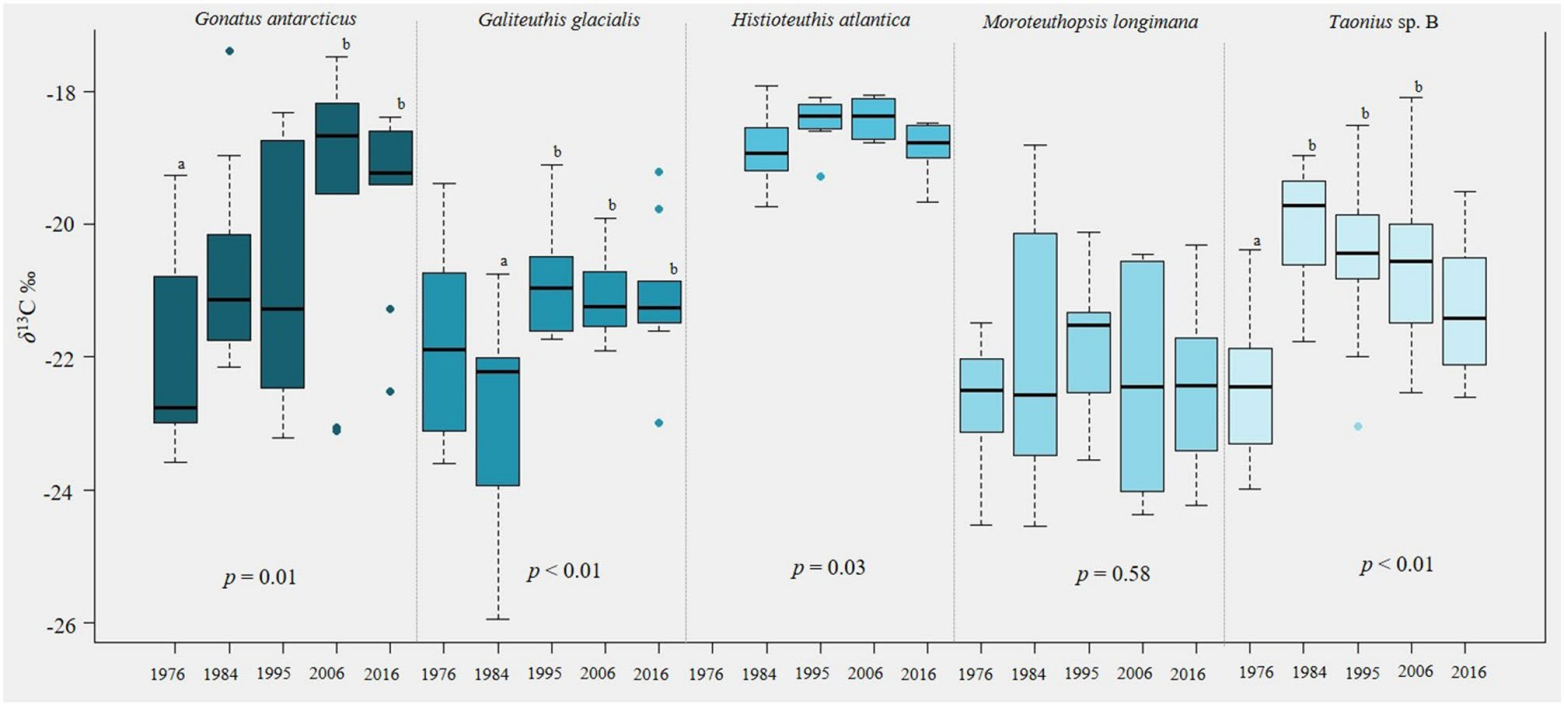
Boxplots of *δ*^13^C values for squid sampled in the southwest Atlantic over five decades. The *p* values are from ANOVAs comparing values between years for each species. The subscripts letters (a vs. b) indicate the years in which the means were significantly different (i.e. the year with “a” is significantly different from the year with “b”) based on multiple comparison tests within species.

**Figure 2:**
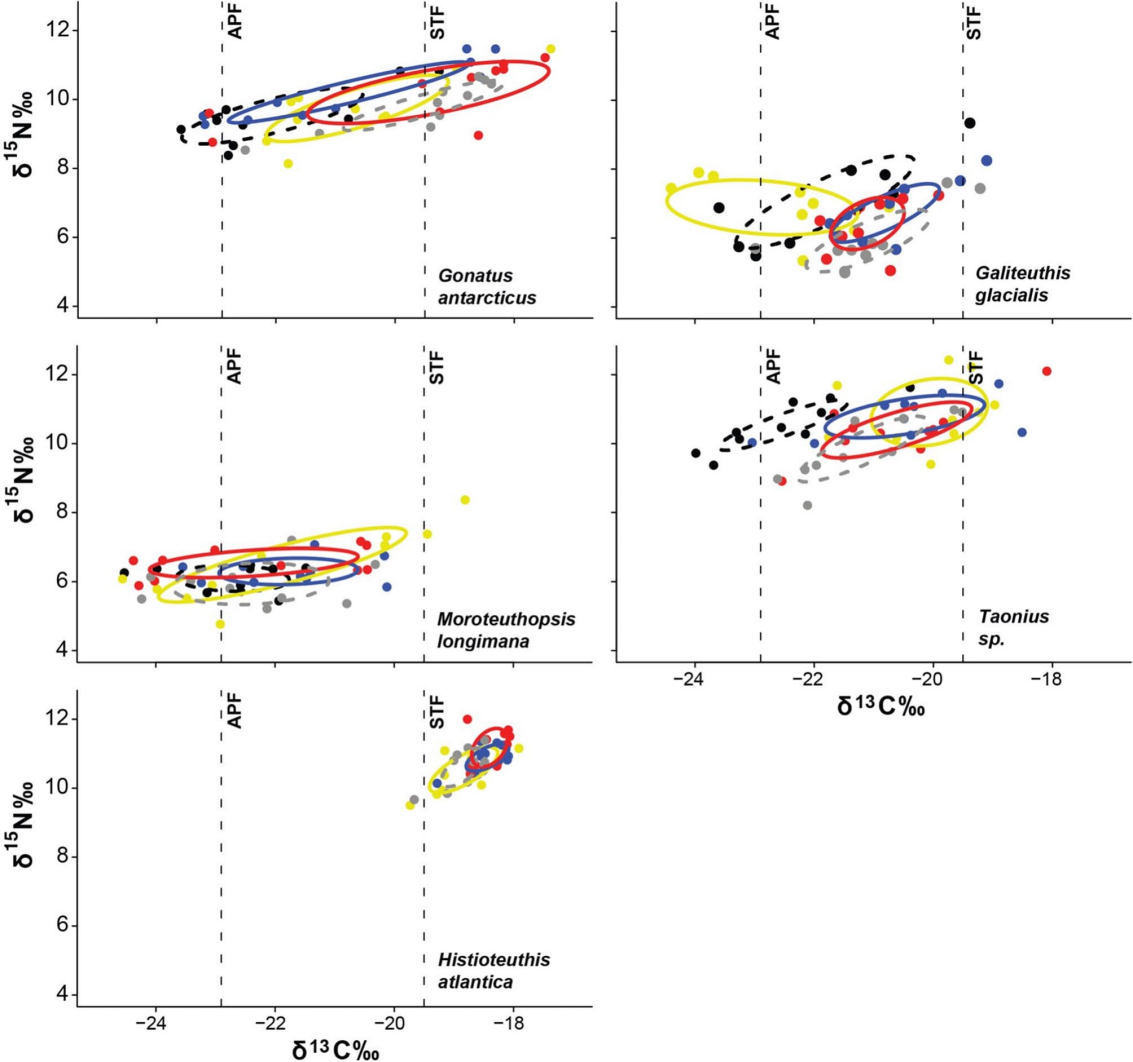
Ecological niches of squid sampled in the southwest Atlantic over five decades. The vertical dashed lines indicate *δ*^13^C values corresponding to—the Antarctic Polar Front (APF) at − 22.9‰ and Sub-tropical Front (STF) at − 19.5‰, and each water masses (Antarctic, subantarctic and subtropical), respectively^67^. The solid or dashed lines represent the standard ellipse areas (SEAc)^75^ for each species. The colours represent the different years: Black-dash—1976; Yellow—1984; Blue—1995; Red—2006 and Dash-grey—2016. R (v. 3.2.5)—SIBER package (https://doi.org/10.1111/j.1365-2656.2011.01806.x).

*δ*^13^C values in beaks ranged from − 17.39‰ in *Gonatus antarcticus* to − 25.95‰ in *Galiteuthis glacialis* (Fig. 1). There were significant differences in mean *δ*^13^C values across decades for four study species, but not in *Moroteuthopsis longimana* (Fig. 1; Supplementary Table S1). However, the multiple comparison test did not identify particular decades that differed significantly in mean *δ*^13^C values in *Histioteuthis atlantica*. Mean *δ*^13^C values were lower in the earlier years (1976 and 1984) for *Taonius* sp. B, *Gonatus antarcticus* and *Galiteuthis glacialis*, suggesting a northward shift in habitat in subsequent decades (2006 and 2016), whereas there was no consistent trend over time for *Histioteuthis atlantica* (Fig. 1). *δ*^15^N values in beaks ranged from 4.77‰ in—*M. longimana* to 12.43‰ in—*Taonius* sp. B (Fig. 2). There were no significant differences across decades in *δ*^15^N values for any of the five squid species, and variation within years was relatively low (SDs ranged from 0.38 to 1.32; Supplementary Table S2).

Based on the Bayesian isotopic niches, all five squid species were distributed mainly in subantarctic waters, with the exception of *H. atlantica*, which occupied subtropical waters (Fig. 2). *Gonatus antarcticus* and *Taonius* sp. B exhibited a wide distribution from subtropical to Antarctic water, throughout the time-series (Fig. 2). The isotopic niche area (SEAc) of most species were wider in 1984, indicating more generalist feeding niches in this particular year (Table 1). A larger SEAc was always associated with expansion of the niche at the limit of the distribution, e.g., to more northerly waters for *Taonius* sp. B, and to more southerly waters for *Galiteuthis glacialis* (Fig. 2).

**Table 1:** Corrected Standard Ellipse Area (SEAc) values for the isotopic niches of squid sampled in the southwest Atlantic over the last five decades (1976–2016).

Species	SEAc				
	1976	1984	1995	2006	2016
*Moroteuthopsis longimana*	1.24	4.07	1.54	2.29	2.71
*Taonius* sp. B (Voss)	1.26	3.24	2.39	2.29	1.86
*Gonatus antarcticus*	2.66	3.04	2.22	4.54	1.53
*Galiteuthis glacialis*	3.78	4.29	1.65	1.47	2.07
*Histioteuthis atlantica*	–	0.90	0.38	0.53	0.52

### Decadal variation in oceanographic conditions

**Figure 3:**
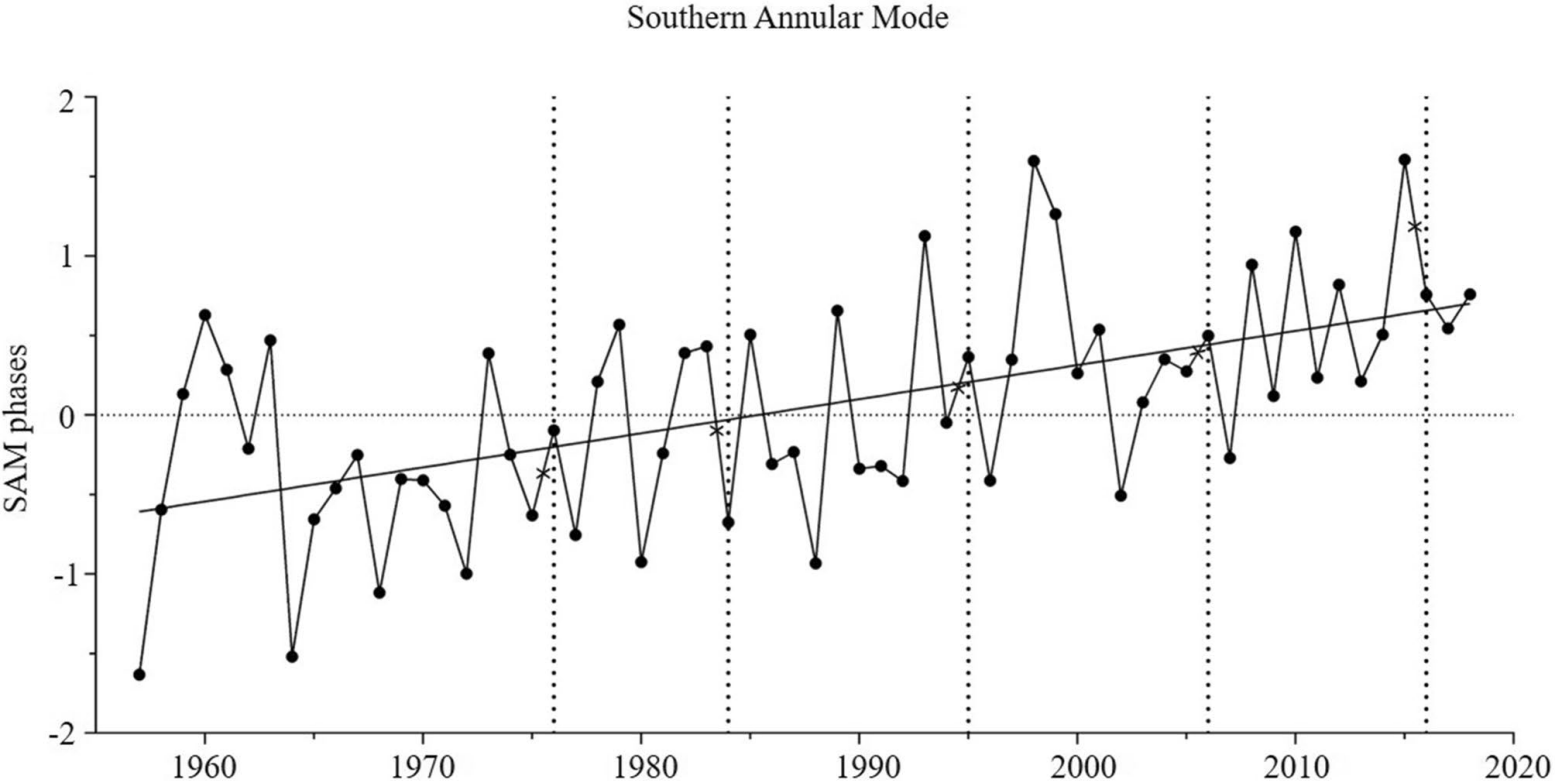
Southern Annular Mode (1957–2018) at Southern Ocean. Points represent the mean of each year. Dashed horizontal lines represent the five sampling years (1976, 1984, 1995, 2006 and 2016) and the cross marks the corresponding lag time used in the analyses.

Lagged values (by 0.5 years) for the Southern Annular Mode (SAM) showed a significant increasing trend in the southwest Atlantic between 1957 and 2018 (general mean = 0.05) (Table 2, Fig. 3). However, 2016 was the only year in which the value for SAM was significantly higher than the long-term mean (t-test: *t*_*11*_ = 3.31, *p* < 0.01) (Table 2). Values for the SAM were mostly positive, and the means did not differ significantly across decades (ANOVA: F_4,55_ = 1.75, *p* = 0.15).

**Table 2:** Differences between the overall mean and values for each sampling year (with the respective time lag), for the environmental indices, SAM (lagged 0.5 years) and SOI (lagged 2 years).

Environmental variables	Years				
	1976	1984	1995	2006	2016
SAM (1957–2018) 0.05 ± 0.69	0.48 ± 0.83	0.25 ± 1.63	-0.03 ± 2.12	0.36 ± 1.53	1.56 ± 1.58
*t* -test	*p* = 0.10	*p* = 0.68	*p* = 0.91	*p* = 0.50	***p < 0.01***
SOI (1951–2018) 0.13 ± 0.58	0.82 ± 0.73	− 1.65 ± 1.27	− 0.52 ± 0.48	− 0.42 ± 0.98	− 0.33 ± 0.58
*t-*test	***p = 0.01***	***p < 0.01***	***p < 0.01***	*p* = 0.07	***p = 0.01***

**Figure 4:**
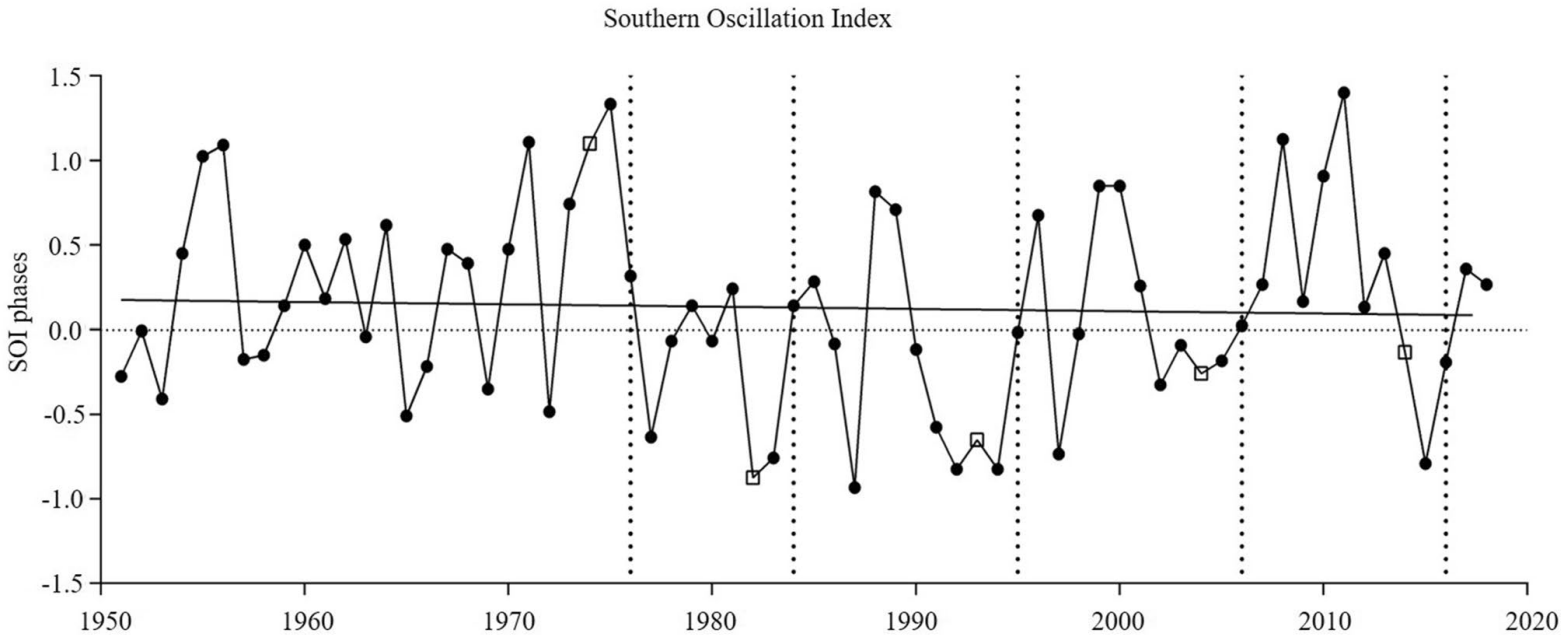
Southern Oscillation Index (1951–2018) at Southern Ocean. Points represent the mean of each year. Dashed horizontal lines represent the five sampling years (1976, 1984, 1995, 2006 and 2016) and the squares the corresponding lag time used in the analyses.

Lagged values (by 2 years) for the Southern Oscillation Index (SOI) showed a weak trend for more negative values over time from 1951 to 2018, indicating more El Niño (negative SOI) than La Niña (positive SOI) phases, particularly in the 1980s (Fig. 4). The lagged values for all study years, except 2006, differed significantly from the long-term mean, and was highest in 1976 (0.82) and lowest in 1984 (− 1.65) (Table 2). Only in one study year, 1976, was there a positive SOI value.

### Beak isotope ratios in relation to the environment

There were significant correlations between the mean isotope ratios and environmental indices only in *Taonius* sp. B, with a significant negative relationship between *δ*^13^C and lagged SOI values (Pearson´s correlation: r = − 0.93, *p* = 0.02), and a negative correlation between *δ*^15^N and lagged SAM values (Pearson’s correlation: r = − 0.92, *p* = 0.03). No other correlation for this species, nor for *Moroteuthopsis longimana*, *Galiteuthis glacialis* or *Gonatus antarcticus* was significant (Supplementary Table S3) (not tested for *Histioteuthis atlantica* because missing data in 1976).

## Discussion

This study provides the first detailed insights into the habitat and trophic level of pelagic nekton in relation to changing environmental conditions in the Southern Ocean over the last five decades. Squid play important role in pelagic food webs in the Southern Ocean, linking lower trophic levels with top predators including fish, marine mammals and seabirds^30,36^. Our analyses of their isotopic niches indicated that the distributions of *Moroteuthopsis longimana*, *Taonius sp*. B (Voss), *Gonatus antarcticus* and *Galiteuthis glacialis* were mainly in subantarctic waters, and of *Histioteuthis atlantica* was in subtropical waters. The distribution of two species—*Gonatus antarcticus* and *Taonius* sp. B—was wide, from subtropical to Antarctic waters, throughout all decades. Most species showed their widest distributions in 1984, which was the sampling year when SOI was lowest (i.e. the strongest El Niño). In contrast, trophic levels of all but one study species have remained broadly stable in the past five decades. Only in *Taonius* sp. B was there a (negative) relationship between *δ*^13^C, *δ*^15^N and one of the environmental indices (SOI or SAM), indicating a change in trophic level as well as habitat.

### Changes in habitat use inferred from *δ*^13^C

To our knowledge this is the first study to examine long-term shifts in habitat of nektonic species in the Southern Ocean ecosystem. The distribution of four of the five squid species changed significantly during the five-decade study, which underlines their capacity to respond and adapt to the characteristics of the surrounding environment. Only *M. longimana* maintained the same habitat use during this time, suggesting a greater tolerance of environmental conditions. Accordingly, our biological sampler, the wandering albatross, has presumably also maintained broadly similar foraging range and habitat preferences over the decades; this is supported by the weaker effect of year than breeding stage or sex on latitude and longitude reached at the furthest point from the colony reached by tracked birds^46^.

In most of the literature, the extent of habitat available for native species (including many endemics) in Antarctic waters was predicted to decline, constraining distributions, whereas lower-latitude species were considered likely to extend their range^34,47,48^. Although most squid in our study appear to have a preference for subantarctic waters^32,43^, given that some individuals had isotopic signatures typical of adjacent water masses, neither the Antarctic Polar Front nor the Subtropical Front (STF) appear to represent major ecological barriers, even though the temperature differential between subtropical and subantarctic waters is substantial (~ 5 °C)^49,50^. Indeed, considering the habitat used by most species extended across three different water masses, some may potentially feed even further north. The exception was the lowest-latitude species, *H. atlantica*, which is unequivocally a warm-water species and inhabited subtropical waters^32,51^. Our results suggest that only for this taxon do cold waters limit distribution, as the isotope analyses suggested that *H. atlantica* only occupied subantarctic waters in the strongest El Niño year in our time series (1984), when waters were warmest in the southwest Atlantic. This species may have an advantage in the future, as predictions from oceanographic models are that warmer water will extend south over the coming decades^34,35^. Maps of the projected anomaly for sea surface temperatures and other oceanographic parameters in the Southern Ocean in 2050–2099 relative to 1956–2005 under different emission scenarios are given in the supplementary material of Freer et al.^23^.

Although there was no consistent long-term trend, based on *δ*^13^C, the changes in habitat of the squid were more marked in the earlier portion of our time series (1970s and 1980s) than in more recent years (2000s and 2010s) (Fig. 1), which may be connected to the intensification of both SOI and SAM^6,10^. The lack of directional shifts suggests that squid respond flexibly to single years (or perhaps short periods) of poor conditions, as in 1984, which was the strongest El Niño of the last five decades^10^. All five squid species appeared to occupy different habitats that year, but the effect was species-dependent; *G. glacialis* moved further south from subantarctic into Antarctic waters, whereas *Taonius* sp. B moved north into the subtropics.

Based on isotopic niches, all five squid species showed a broader range of habitat use or trophic levels in 1984. One explanation is that the El Niño event increased sea surface temperatures, reduced the primary productivity and hence the abundance of Antarctic krill^12–14^. This would explain the shifts in distribution as the squid were forced to become even more generalist and to exploit different size classes or more diverse prey^35,36^, leading to greater niche width. Another non-exclusive explanation based on the SAM index is that there was intensification of the westerly winds, as shown by previous studies, which may affect the distribution of prey if the oceanic fronts moved southwards^7,52^. Nevertheless, as predicted by Rodhouse (2013)^34^, our results demonstrate that there was no effect on squid distribution over the study-period, with the exception of *Taonius* sp. B.

Of all five squid species, only in *Taonius* sp. B were there relationships between distribution, trophic position and the environmental indices. During negative SOI events (El Niño) *Taonius* sp. B occupied more northerly waters, whereas during positive SOI events (La Niña), its distribution shifted southward. This is supported by estimation of its isotopic niche, which in 1976 (high SOI) suggested use of Antarctic waters, and in 1984 (strong El Niño), use of more subtropical waters. On the other hand, SAM had a stronger effect on mean trophic level, which was lower in *Taonius* sp. B during positive SAM, and higher during negative SAM, probably indicating an indirect influence of environmental conditions, as described above.

Changes in distribution of cephalopods in response to environmental conditions, including El Niño events, have been noted in other regions. In north Atlantic and Arctic waters, both pelagic and benthic species (*Todaropsis eblanae*, *Sepietta oweniana*, *Sepiola atlantica*) are now found at much higher latitudes than before, connected to the increasing water temperatures^21,53,54^. Guerra et al. (2002)^55^ also reported the appearance of subtropical species (*Alloteuthis africanus*) in Galician waters attributed to an increase in sea surface temperature. Based on our results, ocean warming seems likely to benefit at least one of our study species, *H. atlantica,* but constrain another, *M. longimana,* the distribution of which is expected to shrink progressively further south.

### Trophic levels based on *δ*^15^N

Our results suggest no substantial changes in trophic level of any of the five study species in the last five decades. However, we recognize the limitations in interpreting *δ*^15^N because of the possibility of shifting baselines or other changes in the trophic web over the long term^56^. These could be overcome in future studies by compound-specific isotopic analysis of amino acids^57,58^. Regardless, squid probably have greater dietary flexibility than many other pelagic taxa, which may help buffer periods of reduced productivity^31,34,51,59^. This might explain why the contribution of each species by number and mass across years in the diet of various top predators has remained remarkably consistent over the last five decades^60–62^. Given the wide and shifting distributions of the study species across different water masses, maintenance of such consistent trophic positions over the last five decades was unexpected, suggesting that their roles in the food web and its general structure changed relatively little.

### Impact of future environmental change on squid in the Southern Ocean

Model predictions for the Southern Ocean point to a continuous warming, and a decrease in sea-ice extent in the study area (southwest Atlantic), as well as changes in wind and ocean currents^3,63,64^. However, squid are characterised by their extreme flexibility and the plasticity of their life-histories^36^, as our results confirm. Previous studies have therefore suggested that squid will probably adapt and thrive in the face of environmental change^34,35^. As squid are short-lived, r-selected species, voracious predators, have high mobility, show rapid turnover of populations and have short generation times, they may adapt more rapidly than long-lived species such as seabirds, marine mammals and many species of fish^3,34^.

According to our study, squid in the Southern Ocean will continue to retain or even increase in their importance in pelagic food webs. All the studied species except *H. atlantica* appeared to exploit a wide range of habitats, while maintaining a similar trophic level over time, despite changing environmental conditions (strong El Niño events, increasing sea surface temperature). Unsurprisingly, *Histioteuthis atlantica*, a subtropical species, should be favoured by ocean warming. Variability in the SAM, as well as in sea-ice extent is therefore unlikely to have major detromental impact on squid species in the Southern Ocean. This agrees with the hypothesis that generalist species with broad dietary niches and habitats are expected to be more resilient to environmental change^65^. Based on our results, ocean warming seems likely to benefit at least one of our study species, *H. atlantica*, but constrain another, *M. longimana*, as mention above. As *H. atlantica* is a warm water species^32^ with an expanding habitat, they may become more available to predators that breed further south, such as wandering albatrosses at South Georgia^39^. Similarly, *M. longimana* which prefers colder waters, might also experience greater predation from penguins, seals, whales and albatrosses if its distribution shifts even further south^30,66^.

As the first study to report long-term changes in nekton in the Southern Ocean, our results for these five squid species provide new clues as to how cephalopod communities may react to a changing environment. This response differed from species to species, regardless of their typical habitat (Antarctic, subantarctic or subtropical waters), or the prevailing environmental conditions. Understanding the scale of the impacts that El Niño might have on each species, and the influence of other environmental or biological drivers on distribution will be key to predicting their future roles in the food web. To conclude, the main cephalopod species in the diet of wandering albatrosses at South Georgia (*M. longimana*, *G. glacialis*, *G. antarcticus*, *H. atlantica* and *Taonius* sp. B) seem to have adapted well to past changes in environmental conditions and this high plasticity may well ensure their continued success.

## Material and methods

### Collection of samples

The cephalopod beaks were extracted mostly from boluses regurgitated naturally by chicks shortly before fledging (October to January), and in one year (i.e. 1984) from chick stomach contents obtained by induced regurgitation. Sampling was at Bird Island, South Georgia (54°00ʹ S, 38°03ʹ W) in 1976, 1984, 1995, 2006 and 2016 (representing each decade from 1970s to present). The boluses were frozen at − 20 °C and analysed at the British Antarctic Survey (BAS), in Cambridge and at Marine Environmental Science Centre (MARE-UC), in Coimbra.

Beaks were cleaned, separated into upper and lower, and then counted following^62^. The upper beaks were not analysed further. Lower beaks were identified using^66^ and checked against a reference collection held at MARE-UC and at BAS. The lower rostral length (LRL) was measured using digital callipers to the nearest 0.01 mm. Mass was estimated using allometric equations in^66^.

### Stable isotope analysis

*δ*^13^C values are a proxy for carbon source (habitat), and in the Southern Ocean present a broad latitudinal gradient, typically decreasing from subtropical waters to the ice edge^43,67^. Carbon isotope ratios show little ^13^C-enrichment with trophic transfer; hence, the values in consumers are thought to closely reflect values at the base of the food web (i.e. baseline), making them useful for studying habitat use^67,68^. *δ*^15^N values reflect trophic position, as consumers are typically enriched ~ 3‰ in ^15^N relative to their prey^68,69^. Ecological applications of stable isotope data, as in this study, requires careful consideration of the spatial–temporal dynamics of isotope values at the baseline^37,70^. Variations in the biological and physical drivers (e.g. CO^2^aq., temperature) will be reflected in upper trophic-level consumers (e.g. cephalopods, fish)^57,70^. It is therefore importance to control for the Suess effect (below), in order to minimize these limitations.

We analysed δ^13^C and δ^15^N in five squid species: *Moroteuthopsis longimana* (former *Kondakovia longimana*), *Taonius* sp. B (Voss), *Gonatus antarcticus*, *Galiteuthis glacialis* and *Histioteuthis atlantica*. The selection was based on two criteria: (1) the five species together form the majority of squid consumed by our biological sampler, the wandering albatross (up to 87.0% of diet by mass)^62^, and (2) together, these five species reflect different water masses (Antarctic, subantarctic and subtropical) in the Southern Ocean^43^, which might show contrasting responses to past environmental changes.

For each year, 10 lower beaks (predominantly adult specimens) were analysed for each of the five cephalopod species. There were no intra-specific differences in LRL between the years, except for *G. glacialis* in 1976, only case when fewer beaks were available (one-way ANOVA: F_4,43_ = 8.38, *p* < 0.01). This minimised possible confounding effects of squid size (and associated age) on differences in stable isotope ratios among years. The entire lower beak was analysed, which provides an integrated, lifetime signal of habitat and trophic level, although biased towards more recent periods when mass increments are greater^37,71^. Beaks were cleaned with 70% ethanol, stored in separated microtubes and dried in an oven. After drying, the beaks were milled using a mixer miller Retsch MM400. *δ*^13^C and *δ*^15^N were determined using a continuous flow isotope ratio mass spectrometer at MARE—Figueira da Foz, following^71^. The results are presented in *δ* notation as deviations from standard references in parts per thousand (‰) according to the following equation:

$$\delta X=\left(\frac{Rsample}{Rstandard}-1\right)\times 1000$$

where X represents ^13^C or ^15^N and Rsample the ratios ^13^C/^12^C or ^15^N/^14^N. Rstandard represents the international reference standard V-PDB (Vienna Pee-Dee Belemnite) and atmospheric N_2_ (AIR) for *δ*^13^C and *δ*^15^N, respectively. Reference material (acetanilide—Thermo) was measured during the analyses to determine internal measurement errors (< 0.1 for *δ*^13^C and < 0.3‰ for *δ*^15^N). The raw isotope data is presented at Supplementary Table S4.

### Environmental data

Two indices of environmental change were used in these analyses. Both affect sea surface temperatures and krill availability in the Scotia Sea region and have documented impacts on demography of albatrosses^11^: the Southern Oscillation index (SOI) (https://www.cpc.ncep.noaa.gov/data/indices/soi) and Southern Annular mode (SAM) (https://www.nerc-bas.ac.uk/icd/gjma/sam.html). Values were standardised, as well as lagged according to the time it takes for a change in the index in the Pacific to affect oceanographic conditions around South Georgia of c. 2 years for SOI^12,13^, and 0.5 years for SAM^11^.

### Data analysis

All statistical tests were performed using α = 0.05 and preceded by a Shappiro-Wilk normality test. Student t-test or ANOVA (normal data) or Mann–Whitney or Kruskal–Wallis (non-normal data) were used to test differences on mean *δ*^13^C and *δ*^15^N between study years, and SAM and SOI versus long-term mean, respectively. Multiple comparisons were performed using Tukey’s or Dunn’s tests. Pearson or Spearman correlations, for normal or non-normal data distributions, respectively, were used to test the correlation between SAM and SOI with stable isotopes (*δ*^13^C and *δ*^15^N) for each species.

Since the Industrial Revolution, the burning of fossil fuels, which are depleted in both ^13^C and ^14^C compared with atmospheric carbon dioxide, has cause an exponential decrease of *δ*^13^C in the biosphere — designated the ''Suess effect''^72^. In addition, the increase in atmospheric CO_2_, and thus aqueous CO_2_, has increased phytoplankton fractionation, reducing *δ*^13^C values^73^. Thus, in our long-term analyses, the measured *δ*^13^C values of squid beaks were adjusted following^73,74^.

*δ*^13^C in the squid beaks were compared with the *δ*^13^C isoscape in the Southern Ocean^67,69^. Threshold, values of − 19.5‰ and − 22.9‰ were considered to represent, respectively, the cut-offs between subtropical and subantarctic waters at the Subtropical Front, and between subantarctic and Antarctic waters at the Antarctic Polar Front (APF) for foraging seabirds^67,69^.

The area of the standard ellipse corrected for small sample size (SEAc) were calculated using the SIBER package for R, and used to compare isotopic niche width, and standard ellipse areas plotted for visualization of the data^75^. Statistical analyses and graphs were made using GraphPad Prism v6.01 and R (v3.2.5)^76^.

### Ethical approval

No live animals were caught for this study. All procedures were performed in accordance with relevant guidelines and regulations for working with live vertebrates and approved by the British Antarctic Survey Animal Welfare and Ethics Board. Permits to handle birds and collect samples were issued by the Government of South Georgia and the South Sandwich Islands. Responsible curators of collections or material owners were participating in the study and thus all necessary permissions were obtained.

## Supplementary information

Supplementary Information
