# Supplementary material for: Long-term changes in habitat and trophic level of Southern Ocean squid in relation to environmental conditions
Source: Sci Rep. 2020 Sep 16;10:15215. doi: 10.1038/s41598-020-72103-6 (PMC7494860; doi:10.1038/s41598-020-72103-6)
Supplement: Supplementary file 1 — Supplementary Information [file 41598_2020_72103_MOESM1_ESM.pdf]

Long-term changes in habitat and trophic level of Southern Ocean squid in relation to environmental conditions

José Abreu[1*], Richard A. Phillips[2], Filipe R. Ceia[1], Louise Ireland[2], Vítor H. Paiva[1], José C. Xavier[1,2]

[1]University of Coimbra, Mare – Marine and Environmental Sciences Centre, Department of Life Sciences, 3000-456 Coimbra, Portugal

[2]British Antarctic Survey, Natural Environment Research Council, High Cross, Madingley Road, Cambridge CB3 0ET, UK

(email: abreu.jose@ua.pt)

Supplementary Information

Table S1. δ^{13}C values (mean ± SD) of squid sampled in the southwest Atlantic over the five-decade study.

Year	δ^{13}C (‰)				
	Moroteuthopsis longimana	*Taonius sp. B*	*Gonatus antarcticus*	*Galiteuthis glacialis*	*Histioteuthis atlantica*
1976	-22.71 ± 0.94	-22.53 ± 1.08[a]	-22.06 ± 1.50[a]	-21.81 ± 1.48	--
1984	-21.88 ± 2.06	-20.07 ± 0.96[b]	-20.63 ± 1.51	-22.87 ± 1.59[a]	-18.84 ± 0.57
1995	-21.77 ± 1.15	-20.48 ± 1.32[b]	-20.78 ± 1.99	-20.81 ± 0.91[b]	-18.44 ± 0.35
2006	-22.36 ± 1.73	-20.61 ± 1.25[b]	-19.44 ± 2.01[b]	-21.10 ± 0.61[b]	-18.40 ± 0.30
2016	-22.41 ± 1.30	-21.20 ± 1.08	-19.52 ± 1.33[b]	-21.09 ± 1.03[b]	-18.84 ± 0.36
Statistics (ANOVA or Kruskal-wallis)	H = 2.89<br>$p = 0.58$	$F_{4,45} = 6.95$<br>$p < 0.01$	H = 13.12<br>$p = 0.01$	$F_{4,43} = 5.06$<br>$p < 0.01$	$F_{3,36} = 3.52$<br>$p = 0.05$
C:N mass ratio	3.49 ± 0.13	3.52 ± 0.11	3.43 ± 0.10	3.63 ± 0.15	3.5 ± 0.08

Table S2. δ^{15}N values (mean ± SD) of squid sampled in the southwest Atlantic over the five-decade study.

Year	δ^{15}N (‰)				
	Moroteuthopsis longimana	*Taonius sp. B*	*Gonatus antarcticus*	*Galiteuthis glacialis*	*Histioteuthis atlantica*
1976	6.1 ± 0.38	10.54 ± 0.72	9.52 ± 0.80	7.05 ± 1.32	--
1984	6.49 ± 1.07	10.91 ± 0.97	9.74 ± 0.95	6.89 ± 0.79	10.53 ± 0.66
1995	6.3 ± 0.38	10.78 ± 0.61	10.21 ± 0.87	6.73 ± 0.84	10.89 ± 0.39
2006	6.54 ± 0.42	10.39 ± 0.80	10.2 ± 0.89	6.42 ± 0.75	11.18 ± 0.55
2016	5.94 ± 0.60	9.84 ± 0.94	9.82 ± 0.72	5.92 ± 0.89	10.6 ± 0.56
Statistics (ANOVA)	$F_{4,45} = 1.65$<br>$p = 0.18$	$F_{4,45} = 2.56$<br>$p = 0.05$	$F_{4,45} = 1.24$<br>$p = 0.31$	$F_{4,43} = 2.21$<br>$p = 0.08$	$F_{3,36} = 2.92$<br>$p = 0.05$
C:N mass ratio	3.49 ± 0.13	3.52 ± 0.11	3.43 ± 0.10	3.63 ± 0.15	3.5 ± 0.08

Table S3. Correlations between beak isotope ratios with the environment conditions (lagged values) of the squid sampled (except *Histioteuthis atlantica*) in the southwest Atlantic, in the five study years. (SOI – Southern Oscillation Index; SAM− Southern Annular Mode).

	$\delta^{13}C$ (carbon isotope)			
	Moroteuthopsis longimana	*Taonius sp. B (Voss)*	*Gonatus antarcticus*	*Galiteuthis glacialis*
SOI	r = -0.44 *p* = 0.46	**r = -0.93 *p* = 0.02**	r = -0.58 *p* = 0.30	r = 0.65 *p* = 0.23
SAM	r = -0.80 *p* = 0.10	r = -0.61 *p* = 0.27	r = 0.28 *p* = 0.65	r = -0.16 *p* = 0.80
	$\delta^{15}N$ (nitrogen isotope)			
SOI	r = -0.58 *p* = 0.31	r = -0.37 *p* = 0.53	r = -0.30 *p* = 0.63	r = 0.08 *p* = 0.90
SAM	r = -0.73 *p* = 0.16	**r = -0.92 *p* = 0.03**	r = -0.33 *p* = 0.59	r = -0.78 *p* = 0.12

Table S4. Raw isotope ($\delta^{13}C$ and $\delta^{15}N$) data of the squid species sampled in southwest Atlantic and respective years.

Year	*Moroteuthopsis longimana*		*Taonius* sp. B (Voss)		*Gonatus antarcticus*		*Galiteuthis glacialis*		*Histioteuthis atlantica*	
	$\delta^{13}C$	$\delta^{15}N$	$\delta^{13}C$	$\delta^{15}N$	$\delta^{13}C$	$\delta^{15}N$	$\delta^{13}C$	$\delta^{15}N$	$\delta^{13}C$	$\delta^{15}N$
1976	-21.461	5.899	-22.188	10.326	-21.873	9.392	-18.267	9.337	–	–
	-20.363	6.397	-20.610	11.325	-21.716	9.704	-21.859	5.483	–	–
	-22.864	6.376	-21.030	10.277	-18.142	10.829	-21.289	5.851	–	–
	-20.817	5.444	-20.761	10.900	-19.663	9.433	-22.478	6.878	–	–
	-21.301	6.376	-22.136	10.130	-21.596	8.663	-20.258	7.965	–	–
	-20.915	6.367	-22.570	9.373	-21680	8.379	-22.145	5.748	–	–
	-21.293	6.483	-19.271	11.630	-18.798	10.824	-19.691	7.837	–	–
	-22.021	5.681	-21.235	11.207	-22.039	9.581	-19.556	7.288	–	–
	-21.456	5.715	-22.869	9.722	-21.436	9.260	–	–	–	–
	-23.410	6.257	-21.433	10.466	-22.472	9.131	–	–	–	–
1984	-23.070	5.785	-18.060	11.121	-20.709	10.050	-19.834	6.899	-18.823	9.502
	-22.008	4.767	-20.850	10.185	-20.727	9.419	-21.276	5.342	-17.638	11.340
	-23.647	6.079	-19.705	10.171	-21.243	8.794	-21.105	7.003	-18.243	11.089
	-18.533	7.369	-19.133	9.399	-20.883	8.138	-21.324	7.332	-18.250	10.379
	-19.220	7.294	-18.329	10.907	-19.757	9.730	-23.488	7.442	-17.001	11.154
	-17.898	8.366	-18.745	10.287	-19.295	9.461	-23.026	7.900	-18.288	9.938
	-21.316	6.729	-18.831	12.426	-18.060	10.910	-22.781	7.793	-18.377	9.822
	-19.248	7.051	-18.770	10.683	-20.836	9.937	-25.040	6.281	-17.225	11.092
	-22.569	5.522	-18.446	12.227	-16.477	11.469	-20.413	6.222	-17.801	10.862
	-22.145	5.899	-20.703	11.686	-19.248	9.519	-21.290	6.681	-17.628	10.092
1995	-19.498	5.847	-18.276	11.737	-18.173	11.469	-20.113	7.005	-17.655	11.312
	-20.949	6.159	-19.753	10.243	-20.928	9.545	-21.113	6.418	-17.572	11.251
	-22.923	6.429	-22.417	10.028	-21.844	9.404	-19.862	7.417	-17.631	10.778
	-22.611	5.969	-17.887	10.326	-20.373	9.739	-18.479	8.247	-17.982	10.388
	-21.911	6.442	-19.700	11.079	-17.691	11.465	-20.829	6.676	-17.931	11.357
	-21.730	5.980	-19.856	11.144	-17.944	10.652	-20.558	5.886	-17.853	11.002
	-20.710	7.066	-19.226	11.463	-22.596	9.519	-20.979	6.078	-17.918	10.922
	-19.539	6.751	-21.371	10.003	-21.353	9.915	-21.025	6.217	-18.658	10.140
	-20.847	6.091	-20.192	11.105	-18.108	11.083	-20.002	5.669	-17.482	10.820
	-20.781	6.261	-19.870	10.720	-22.567	9.275	-18.922	7.663	-17.463	10.928
2006	-23.946	5.886	-17.757	12.102	-19.207	10.459	-20.919	6.147	-17.751	11.687
	-23.546	6.621	-19.875	9.846	-17.972	10.834	-20.953	6.812	-17.940	10.642
	-22.671	6.910	-19.662	10.406	-17.842	10.880	-19.571	7.241	-18.388	10.412
	-23.682	6.020	-21.142	10.083	-17.145	11.214	-21.563	6.501	-18.436	11.998
	-20.275	6.332	-20.552	10.303	-17.842	11.035	-20.559	6.985	-18.403	10.594
	-20.123	7.053	-21.013	10.453	-18.264	8.959	-20.381	5.056	-17.770	11.272
	-20.222	7.161	-21.327	10.869	-22.725	8.760	-20.173	7.144	-17.725	11.503
	-21.560	6.468	-19.741	10.358	-18.380	10.638	-21.195	6.048	-17.814	11.582
	-20.112	6.349	-19.493	10.617	-18.910	9.638	-20.895	6.909	-18.302	10.672
	-24.037	6.612	-22.204	8.910	-22.777	9.602	-21.453	5.388	-18.117	11.410
2016	-21.641	7.195	-22.536	8.972	-18.523	10.668	-21.055	5.499	-18.693	10.177
	-20.239	6.502	-20.617	9.770	-18.415	10.567	-21.523	5.643	-18.407	10.752
	-24.007	6.142	-22.077	9.240	-19.211	9.911	-21.407	4.990	-18.923	10.806
	-21.809	5.530	-21.886	9.370	-18.706	10.115	-19.690	7.605	-18.432	10.500
	-24.159	5.499	-19.438	10.914	-19.119	10.224	-21.293	5.652	-18.592	10.768
	-20.716	5.365	-19.573	10.980	-19.324	9.203	-21.409	5.031	-18.685	11.168
	-22.679	5.807	-20.425	10.730	-22.436	8.533	-19.136	7.439	-18.868	10.963
	-23.336	6.021	-22.034	8.208	-19.173	9.541	-22.906	5.697	-19.034	9.846
	-22.637	6.127	-21.239	10.659	-18.306	10.448	-20.772	5.804	-18.405	11.389
	-22.056	5.214	-21.435	9.594	-21.190	9.017	-20.941	5.840	-19.588	9.669
